# Spatial analysis of cholangiocarcinoma in relation to diabetes mellitus and *Opisthorchis viverrini* infection in Northeast Thailand

**DOI:** 10.1038/s41598-024-61282-1

**Published:** 2024-05-07

**Authors:** Kavin Thinkhamrop, Kulwadee Suwannatrai, Matthew Kelly, Apiporn T. Suwannatrai

**Affiliations:** 1 Cholangiocarcinoma Screening and Care Program (CASCAP), Faculty of Medicine, Khon Kaen University, Khon Kaen, Thailand; 2 Cholangiocarcinoma Research Institute (CARI), Khon Kaen, Thailand; 3 Health and Epidemiology Geoinformatics Research (HEGER), Faculty of Public Health, Khon Kaen University, Khon Kaen, Thailand; 4 Faculty of Science and Technology, Sakon Nakhon Rajabhat University, Sakon Nakhon, Thailand; 5 National Centre for Epidemiology and Population Health, College of Health and Medicine, Australian National University, Canberra, Australia; 6 Department of Parasitology, Faculty of Medicine, Khon Kaen University, Khon Kaen, Thailand

**Keywords:** Cancer screening, Risk factors, Disease prevention

## Abstract

Cholangiocarcinoma (CCA) exhibits a heightened incidence in regions with a high prevalence of *Opisthorchis viverrini* infection, with previous studies suggesting an association with diabetes mellitus (DM). Our study aimed to investigate the spatial distribution of CCA in relation to *O. viverrini* infection and DM within high-risk populations in Northeast Thailand. Participants from 20 provinces underwent CCA screening through the Cholangiocarcinoma Screening and Care Program between 2013 and 2019. Health questionnaires collected data on *O. viverrini* infection and DM, while ultrasonography confirmed CCA diagnoses through histopathology. Multiple zero-inflated Poisson regression, accounting for covariates like age and gender, assessed associations of *O. viverrini* infection and DM with CCA. Bayesian spatial analysis methods explored spatial relationships. Among 263,588 participants, *O. viverrini* infection, DM, and CCA prevalence were 32.37%, 8.22%, and 0.36%, respectively. The raw standardized morbidity ratios for CCA was notably elevated in the Northeast’s lower and upper regions. Coexistence of *O. viverrini* infection and DM correlated with CCA, particularly in males and those aged over 60 years, with a distribution along the Chi, Mun, and Songkhram Rivers. Our findings emphasize the association of the spatial distribution of *O. viverrini* infection and DM with high-risk CCA areas in Northeast Thailand. Thus, prioritizing CCA screening in regions with elevated *O. viverrini* infection and DM prevalence is recommended.

## Introduction

Cholangiocarcinoma (CCA), a form of cancer impacting the bile ducts, exhibits a noteworthy prevalence in Thailand. Recent investigations indicate that Thailand has the highest recorded incidence of CCA globally. Specifically, the age-standardized rate (ASR) is reported at 33.4 per 100,000 in men and 12.3 per 100,000 in women^1,2^. The disease burden associated with intrahepatic CCA in Thailand is also substantial, with an incidence rate of 14.6 per 100,000 population per year during the period 2009—2013^3^. This elevated incidence in Thailand is closely tied to the widespread occurrence of *Opisthorchis viverrini* infection^4–7^. *Opisthorchis viverrini* poses a significant public health challenge, with a documented prevalence in Thailand, Laos, Cambodia, Myanmar, and Vietnam^8,9^. The primary mode of infection stems from the consumption of raw or undercooked freshwater cyprinid fish^1,9–11^. This dietary practice is deeply embedded in the cultural norms of the people in northeastern Thailand and the broader Lower Mekong Region^12^. Previous studies conducted in northeastern Thailand revealed that the prevalence of *O. viverrini* infection was estimated at 17% in 2009^9^. A subsequent study in 2014 reported an increase to about 23%, with a higher prevalence observed among males and individuals aged 40–49 years^13^. Moreover, following the treatment of *O. viverrini* infections, reinfection rates were approximately 10%, attributed to unchanged dietary habits^14^. This parasitic infection triggers chronic inflammation, resulting in oxidative DNA damage to the infected biliary epithelium and subsequent malignant transformation^1^. Notably, recent investigations have indicated a declining trend in the incidence of CCA in Thailand. Both males and females have experienced a significant reduction in the incidence rate, marking a positive shift in the epidemiological landscape^15^.

The incidence of CCA exhibits significant regional variability, with distinct risk factors identified across diverse populations. Previous studies in Thailand have outlined specific risk factors for CCA, including *O. viverrini* infection, the consumption of raw cyprinid fish, a family history of cancer, the use of praziquantel (PZQ), hepatitis C virus, hepatitis B virus, cirrhosis, obesity, alcohol consumption, tobacco smoking, and diabetes mellitus (DM)^2,16,17^. Furthermore, a recent study conducted in northeastern Thailand has shed light on an intriguing correlation. This research uncovered that individual infected with *O. viverrini* not only faced an increased risk of CCA but that this risk was further amplified in individuals who also had DM^18^. This dual association suggests a potential synergistic effect between *O. viverrini* infection and DM in elevating the susceptibility to CCA.

While prior research has explored the association of various factors with the development of CCA, a noteworthy revelation pertains to the connection between the combined presence of *O. viverrini* infection and DM and the development of CCA. Despite these insights, the scarcity of spatial analyses aimed at pinpointing geographic areas with a high prevalence of CCA, while considering associated factors, remains evident. This gap is particularly pronounced in regions characterized by a heightened prevalence of both *O. viverrini* infection and DM.

Extensive research has delved into the spatial analysis of CCA concerning *O. viverrini* infection and DM in Northeast Thailand. The heightened prevalence of liver and biliary tract abnormalities in this region, predominantly attributed to the distribution of *O. viverrini*, has been a focal point of spatial analysis studies^19,20^. These investigations aim to unravel the geographical distribution and clustering of CCA cases in Northeast Thailand, offering valuable insights into the environmental and regional factors contributing to the CCA prevalence^20^. These studies have played a pivotal role in emphasizing the pressing need for eradicating the liver fluke and identifying high-risk populations to effectively address the elevated incidence of CCA in the region^20^. Additionally, the investigation into the correlation between DM and CCA has garnered attention, as DM is considered a potential risk factor for CCA^18,21–23^. Previous studies have suggested that individuals with DM were more likely to be incident cases of CCA^24^, and another study demonstrated an association between DM and shorter survival among CCA patients^25^. Understanding the spatial distribution of CCA cases in relation to DM and *O. viverrini* infection is paramount for crafting targeted prevention and control strategies. Moreover, this knowledge is crucial for enhancing early detection and treatment outcomes, particularly in high-risk areas such as Northeast Thailand^18,26^. This region is endemic for *O. viverrini*, the primary cause of abnormalities in the biliary system examined in this study and a key factor on the etiological pathway leading to the development of CCA^27^.

Geographic Information System (GIS) analysis has emerged as a prevalent tool in epidemiological research, facilitating the identification of disease clustering, spatial patterns, and the analysis of interactions between environmental factors and health. Nevertheless, the authors are not aware of any studies employing these methods in the investigation of the distribution of CCA, DM, and the history of *O. viverrini* infection. Therefore, our study aimed to explore the spatial distribution of CCA in relation to *O. viverrini* infection and DM within high-risk populations in Northeast Thailand.

## Materials and methods

### Study area

**Figure 1:**
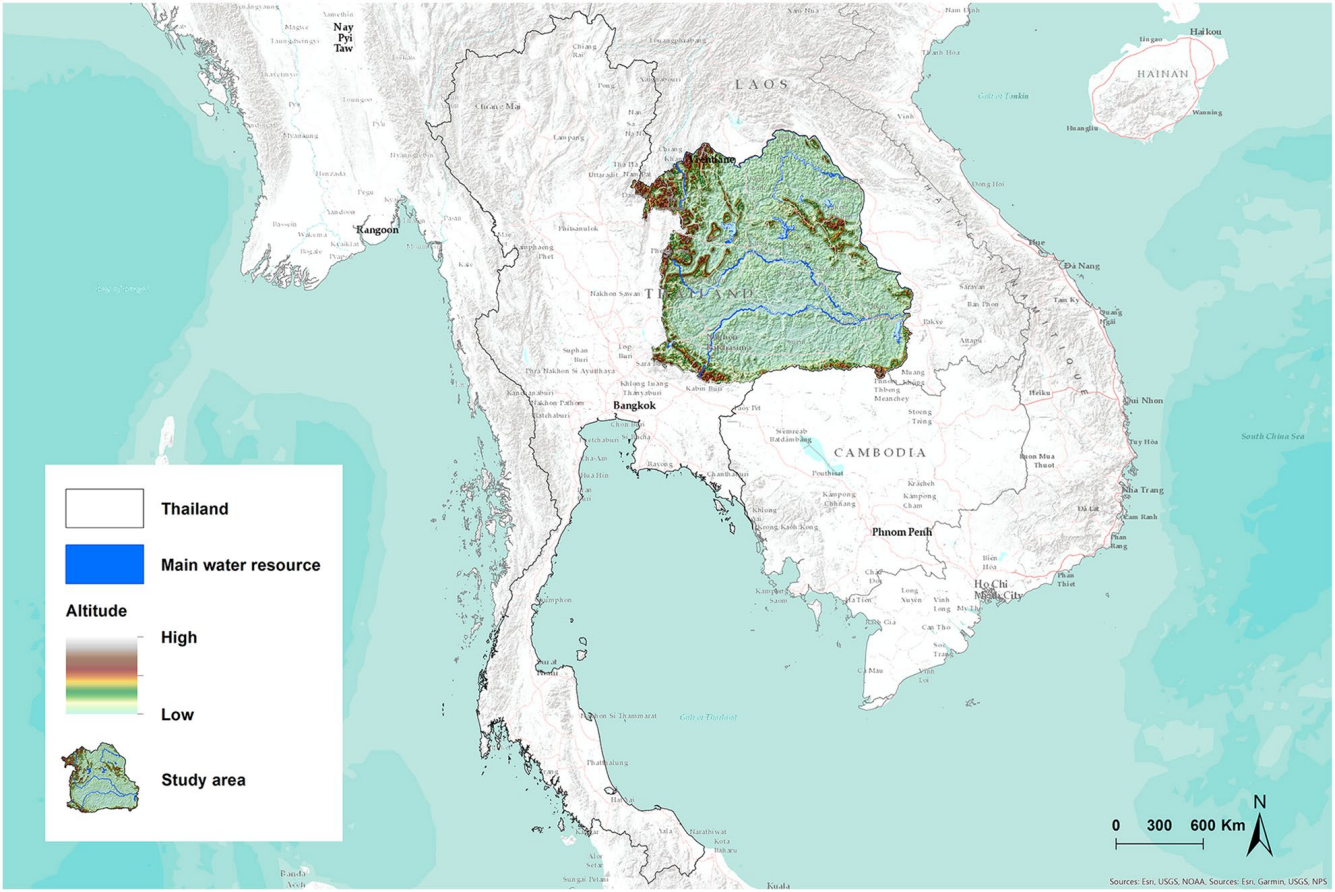
Map of the study area. Map was created using ArcGIS Pro software version 3.2 (ESRI: https://www.esri.com/en-us/home).

The study was conducted in Northeast Thailand, situated between latitudes 14.50°N and 17.50°N, and longitudes 102.12°E and 104.90°E, encompassing an approximate area of 168,854 km^2^ (Fig. 1). This region comprises 20 provinces (Supplementary Fig. 1), and data for this study was systematically gathered from all 2678 sub-districts distributed across these provinces.

### Study design and data sources

The cross-sectional study was reported in accordance with the Strengthening the Reporting of Observational Studies in Epidemiology (STROBE) statement. The data for this study were obtained from the Cholangiocarcinoma Screening and Care Program (CASCAP) in northeastern Thailand. CASCAP represents the pioneering initiative for CCA screening within a high-risk population, employing a community-based, bottom-up approach^27^. The CASCAP screening program endeavors to encompass all residents of northeastern Thailand, achieving this through various methods and settings, such as tertiary-care hospitals, district-level hospitals, and mobile screening sessions at the sub-district level. For this study, we included all participants who underwent CCA screening and were subsequently enrolled in the CASCAP database.

### Study population

Northeastern Thailand boasts a population of approximately 21 million individuals, accounting for roughly one-third of Thailand's total population. Our study encompasses all individuals who participated in CASCAP across 20 provinces and received information regarding their history of *O. viverrini* infection and diagnosis of DM. Specifically, our study sample comprises individuals who were enrolled in the CASCAP database between 2013 and 2019, totaling 263,588 subjects. Population data for the sub-districts during the study period (2013–2019) were acquired from the Thailand Official Statistics Registration Systems website (https://stat.bora.dopa.go.th).

### Study outcome and independent variables

The primary outcome in this study was the diagnosis of CCA, which was determined through a combination of ultrasonography (US), computed tomography (CT)/magnetic resonance imaging (MRI), and histopathological examination. Positive US findings were defined as those indicating liver masses and/or bile duct dilatation, indicative of suspected CCA. Individuals falling into this category were subsequently referred for either CT or MRI scans. The results of the CT/MRI scans were categorized as either positive or negative for CCA. For confirmation of positive CCA cases, biopsy procedures were performed. Participants who did not exhibit suspicious findings on US, CT/MRI scans, or had histologically negative results were classified as negative for CCA. The diagnosis of CCA was only confirmed through biopsy results. The independent variables in this study encompassed the key factors of interest, namely the history of *O. viverrini* infection and the diagnosis of DM, both of which were categorized into two groups (negative/positive). Additionally, the covariates considered for analysis consisted of gender and age at the time of enrollment. These factors were obtained through interviews with study participants using the CASCAP questionnaires.

### Statistical analysis

The prevalence of CCA, *O. viverrini* infection, and DM was calculated as percentages, with the number of positive cases as the numerator and the total number of participants as the denominator. In order to assess differences in the prevalence of CCA across different categorical factors, we utilized the chi-square test statistic, employing a significance level of 0.05. The prevalence is reported along with its corresponding 95% confidence interval (CI). This CI is determined by calculating the lower bound as the proportion value minus the product of the z-score for the 95% CI (1.96) and the standard error (SE). The SE is computed as the square root of the proportion value multiplied by one minus the proportion value, divided by the number of samples. Subsequently, the upper bound is obtained by the proportion value plus the product of the z-score and the SE. These values are then multiplied by 100 and presented as the 95% CI of the prevalence. All statistical analyses were conducted using STATA version 18 (StataCorp, College Station, TX, USA).

We accounted for differences in population sizes by calculating raw standardized morbidity ratios (SMR) for the overall positive of CCA in each sub-district of Northeast Thailand from 2013 to 2019, using the following formula:

$$Y_{i} = ~\frac{{O_{i} }}{{E_{i} }}$$

where *Y*_*i*_ represents the SMR in sub-district *i*, *O*_*i*_ stands for the observed number of cases for CCA in the sub-district, and *E*_*i*_ represents the expected number of cases in the sub-district over the study period. We calculated the expected number of cases by multiplying the population of each sub-district by the overall crude rates of these conditions during the study period^28^.

### Spatial analysis

This analysis covered a region that included 2678 sub-districts in Northeast Thailand. The administrative boundaries for these sub-districts were defined using polygon shapefiles obtained from the DIVA-GIS website (www.diva-gis.org). The spatial dataset, which included SMR for CCA were imported into ArcGIS Pro software version 3.2 (ESRI Inc., Redlands, CA, USA) and projected onto the Universal Transverse Mercator (UTM) coordinate system zone 48 N. The polyline shapefiles representing main water resources in Northeast Thailand were obtained from the DIVA-GIS website (www.diva-gis.org). The administrative boundary map encompassed a total of 2678 sub-district-level areas^20^.

We identified cases where *O. viverrini* infection history was present alongside CCA or DM, cases where DM co-occurred with CCA, and cases where all three conditions coexisted as OVCCA, DMCCA, and OVDMCCA, respectively. These cases, along with the spatial distributions of demographic data, were integrated into ArcGIS Pro software. The datasets were geospatially linked based on the administrative boundary map of Northeast Thailand. This allowed us to aggregate and extract data by sub-district areas and create variables for subsequent statistical analyses. The process of linking and analyzing the data followed established procedures previously employed in spatial analyses within this region^20^. The cut-off points reported in the figure legends were determined using the Natural Breaks (Jenks) classification method in the ArcGIS Pro software.

We initially conducted bivariate Poisson regression analyses to explore associations between CCA cases and various independent variables, including gender, age group, history of *O. viverrini* infection and DM. This step aimed to identify covariates supported by evidence from prior articles, indicating associations with the development of CCA or demonstrating significant relationships with CCA prevalence (with a threshold of *P* value < 0.25) in the bivariate models. Variables meeting this criterion were subsequently incorporated into the final models. To assess collinearity among all included variables, we performed Pearson correlation analyses. All preliminary statistical analyses were carried out using STATA software version 18.0 (Stata Corporation, College Station, TX, USA).

### Spatial poisson regression analysis

We conducted a Spatial Poisson regression analysis in our study. To address the presence of zero cases in some sub-districts for the CCA, we opted for a zero-inflated Poisson (ZIP) regression model rather than a standard Poisson regression. The ZIP regression was implemented using a Bayesian framework in WinBUGS software version 1.4.3, developed by the Medical Research Council in Cambridge, UK. Three distinct models were constructed to account for different sources of variability. The first model, referred to as Model I, incorporated unstructured random effects for gender, age groups, history of *O. viverrini* infection, and DM. In the second model, Model II, we introduced spatially structured random effects alongside the same covariates as Model I. Finally, the third model, Model III, was a convolution model that included both spatially structured and unstructured random effects, while maintaining the same covariate set as the previous two models.

The convolution model, with an outcome of observed cases of CCA (numbers), Y, for the *i*th sub-district in Northeast Thailand (i = 1 to 2678), for the *j*th gender group and *k*th age group was structured as follows:

$$P({Y}_{ijk}= {y}_{ijk})=\left\{\begin{array}{c}\omega +1 \left(1-\omega \right){e}^{-\mu }, {y}_{ijk}=0\\ \left(1-\omega \right){e}^{-\mu } {\mu }_{ijk}^{{y}_{ijk}}/{y}_{ijk}, {y}_{ijk}>0;\end{array}\right.$$

$$Y_{{ijk}} \sim ~{\text{Poisson}}(\mu _{{ijk}} )$$

$${\text{log}}(\mu_{ijk} ) \, = {\text{log}}({\text{E}}_{ijk} ) \, + \theta_{ijk}$$

$$\begin{array}{*{20}c} {\theta_{ijk} = \alpha + \beta_{{1}} \times {\text{gender}}_{j} + \beta_{{2}} \times {\text{age}}_{k} + \beta_{{3}} \times O.} & {Viverrini\;{\text{infection}}_{i} + \beta_{{4}} \times {\text{ DM}}_{i} + {\text{u}}_{i} + {\text{s}}_{i} } \\ \end{array}$$

where E_*ijk*_ is the expected number of CCA cases (offsetting population size) in sub-district *i*, gender *j*, age group *k*, μ_*ijk*_ is the parameter of distribution, θ_*ijk*_ is the mean log relative risk (RR) and ω ∈ [0,1] relates to proportion of extra zero; α is the intercept, and *β*_1_*, β*_2_*, β*_3*,*_ and *β*_4_ are the coefficients for age (≤ 60 years as the reference category), gender (female as the reference category), *O. viverrini* infection history, DM; u_*i*_ and s_*i*_ are the unstructured and structured random effects (assuming a mean of zero and variances of σ_u_^2^ and σ_s_^2^ respectively).

In the modelling the spatially structured random effects, we implemented a conditional autoregressive (CAR) prior structure. This structure was employed to account for spatial relationships among sub-districts within the study area. To represent these relationships, we utilized an adjacency weights matrix, where a weight of 1 denoted sub-districts sharing a border, and 0 indicated no shared border. For the model’s priors, we employed a flat distribution for the intercept and a normal distribution for the coefficients, with a mean of zero and a precision (the inverse of variance) set at 0.0001^20^. The precision of both unstructured and spatially structured random effects was modelled using non-informative gamma distributions, characterized by shape and scale parameters of 0.001. These methodologies have previously been applied in spatial analyses of CCA in Northeast Thailand.

We initiated the analysis by discarding the initial 10,000 iterations as a burn-in period. Subsequently, we executed sets of 15,000 iterations and scrutinized them for signs of convergence in the Monte Carlo chains. This process was iterated three times, with the outcomes of the first two iterations being discarded. At this juncture, we conducted a visual examination of the posterior density plots to ascertain that convergence had indeed been achieved. The final model selection hinged on striking a balance between model fit and parsimony. We employed the deviance information criterion (DIC) to guide our decision-making, ultimately selecting the model that yielded the lowest DIC score. To establish statistical significance of the covariates, we set an α-level of 0.05, determined by analyzing the 95% credible intervals (CrI) for mean posterior relative risks (RRs). Variables were considered statistically significant if the 95% CrI for the RRs did not encompass 1^20,29^. Finally, to visually represent the spatial distribution of posterior means for both unstructured and structured random effects, we utilized ArcGIS Pro software to generate maps.

### Ethical considerations

The research protocol was approved by Khon Kaen University Ethics Committee for Human Research, reference number HE631061. The data were provided from the CASCAP. The CASCAP data collection was conducted according to the principles of Good Clinical Practice, the Declaration of Helsinki, and national laws and regulations about clinical studies. It was approved by the Khon Kaen University Ethics Committee for Human Research under the reference number HE551404. All patients gave written informed consent for the study.

## Results

### Descriptive summary

A total of 263,588 participants undergoing screening for CCA were included in our study. Approximately two-thirds of the participants were females (60.7%), with the majority being aged 60 years or less, reflecting a mean age of 55.7 (standard deviation = 9.2) years (Table 1). The prevalence of *O. viverrini* infection was 32.37% (95% CI: 32.19–32.54), and the prevalence of those diagnosed with DM was 8.22% (95% CI: 8.12–8.33).

**Table 1:** Baseline characteristics and health history of participants in the CASCAP study 2013–2019

Characteristics	Frequency	Percentage
Gender		
Female	160,007	60.70
Male	103,581	39.30
Age groups (years)		
≤ 60 years	185,015	70.19
> 60 years	78,573	29.81
Mean (standard deviation)	55.7 (9.2)	
*O. viverrini* infection		
No	178,275	67.63
Yes	85,313	32.37
Diabetes mellitus		
No	241,917	91.78
Yes	21,671	8.22

Percentage—The percentage per 100.

### Distribution of cholangiocarcinoma

Table 2 illustrates the prevalence of CCA among a total of 263,588 participants, with 0.36% diagnosed with CCA. Elevated prevalence was observed in individuals aged over 60 years (0.66%; 95% CI: 0.60–0.72), those diagnosed with DM (0.60%; 95% CI: 0.49–0.70), males (0.59%; 95% CI: 0.54–0.63), and those infected with *O. viverrini* (0.47%; 95% CI: 0.42–0.51), respectively.

**Table 2:** Prevalence of cholangiocarcinoma for each factor.

Factors	Participants	Cholangiocarcinoma			
		Number of cases	Prevalence	95% CI	*P* value
Overall	263,588	944	0.36	N/A	N/A
Gender					< 0.001
Female	160,007	336	0.21	0.19–0.23	
Male	103,581	608	0.59	0.54–0.63	
Age groups					< 0.001
≤ 60 years	185,015	426	0.23	0.21–0.25	
> 60 years	78,573	518	0.66	0.60–0.72	
*O. viverrini *infection					< 0.001
No	178,275	544	0.31	0.28–0.33	
Yes	85,313	400	0.47	0.42–0.51	
Diabetes mellitus					< 0.001
No	241,917	815	0.34	0.31–0.36	
Yes	21,671	129	0.60	0.49–0.70	

N/A, Not applicable; 95% CI, 95% Confidence interval of the prevalence; Prevalence, The prevalence per 100; *P* value, Probability values from chi-square tests.

**Figure 2:**
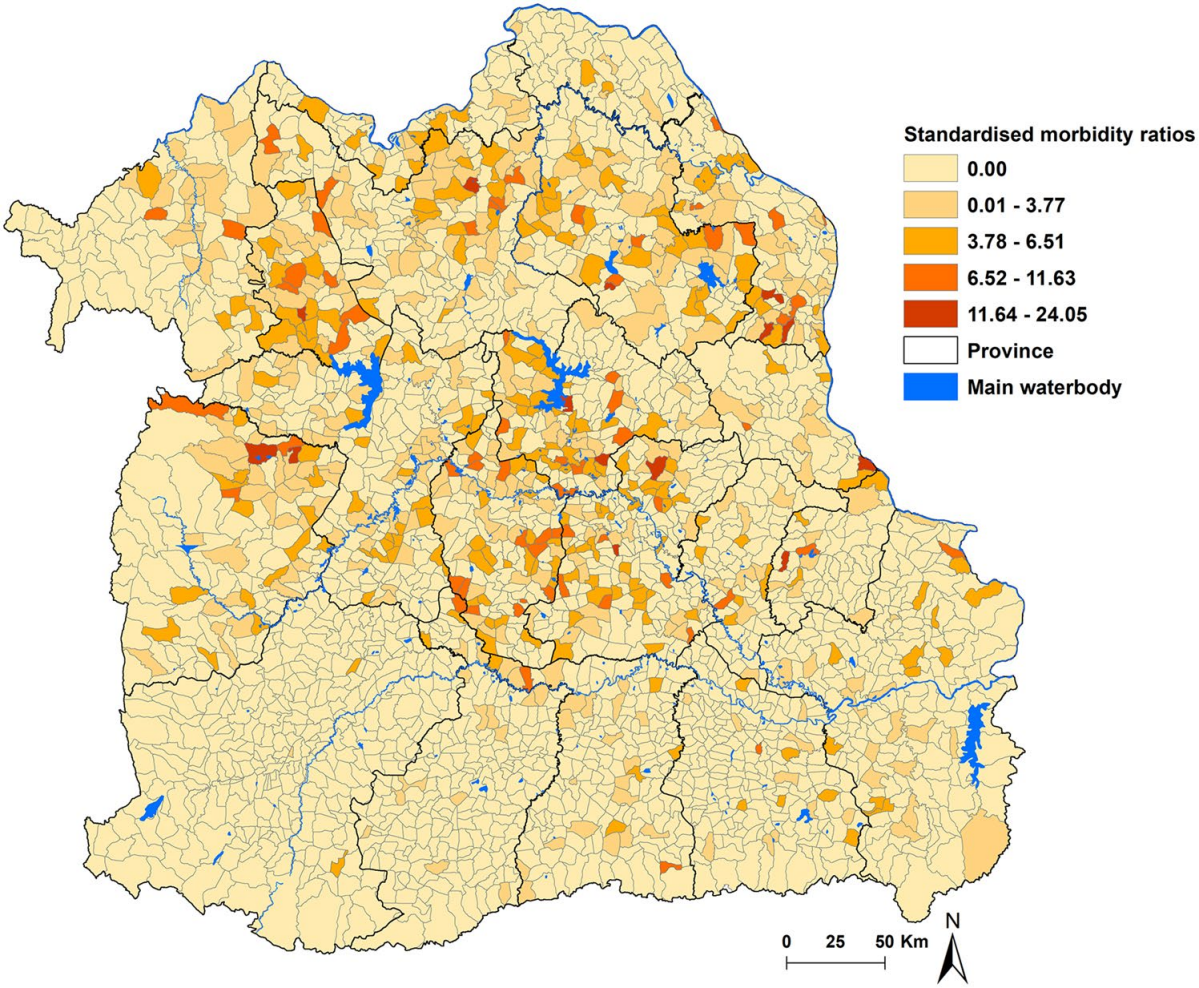
Raw standardized morbidity ratios of cholangiocarcinoma by sub-districts in Northeast Thailand. Map was created using ArcGIS Pro software version 3.2 (ESRI: https://www.esri.com/en-us/home).

Over the seven-year period under examination, the overall crude incidence rate of CCA was calculated to be 2.64 cases per 100,000 population. At the sub-district level, the incidence rates exhibited considerable variation, spanning from 0 to 38 cases per 100,000 population. Particularly noteworthy were the substantial spatial disparities observed in the incidence rate of CCA among different sub-districts. Figure 2 visually depicts the spatial distribution of SMR for CCA in Northeast Thailand at the sub-district level.

**Figure 3:**
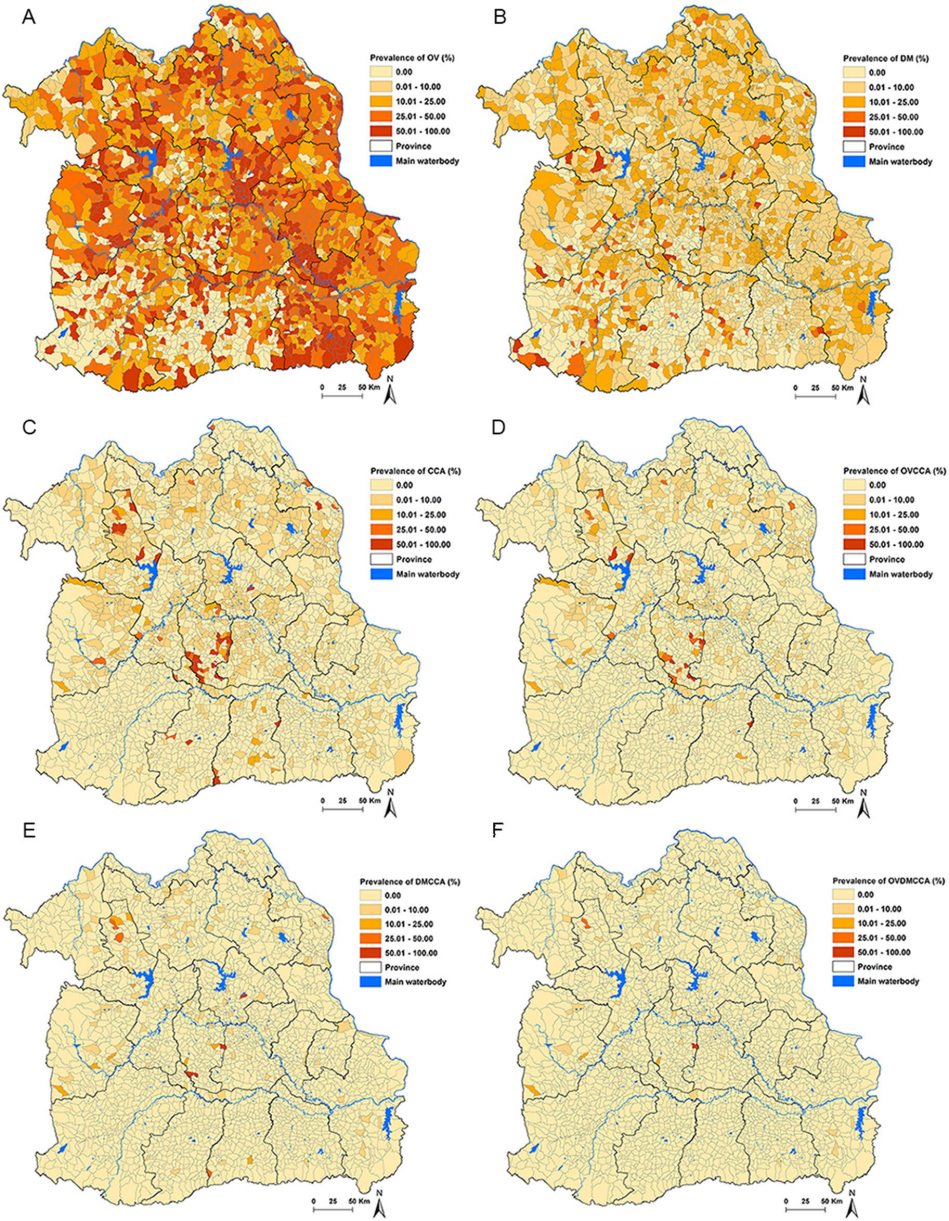
Prevalence of: (**A**) *Opisthorchis viverrini* infection (**B**) diabetes mellitus (**C**) cholangiocarcinoma (**D**) *O. viverrini* + cholangiocarcinoma (**E**) diabetes mellitus + cholangiocarcinoma and (**F**) *O. viverrini* + diabetes mellitus + cholangiocarcinoma. *OV* *Opisthorchis viverrini*; *DM* Diabetes mellitus, *CCA* Cholangiocarcinoma*.* Maps were created using ArcGIS Pro software version 3.2 (ESRI: https://www.esri.com/en-us/home).

Figures 3 depict the distribution of prevalence for *O. viverrini* infection history, DM, CCA, OVCCA, OVDM, DMCCA, and OVDMCCA within the Northeast Thailand. The spatial representation highlights Chaiyaphum, Khon Kaen, Kalasin, Yasothon, and Sisaket Provinces as having the highest prevalence of *O. viverrini* (Fig. 3A). Similarly, the prevalence of DM is most pronounced in Nakhon Ratchasima, Khon Kaen, Maha Sarakham, Surin, Ubon Ratchathani, Kalasin, Nakhon Phanom, Bueng Kan, and Nong Khai Provinces (Fig. 3B). Concerning CCA, Nong Bua Lamphu, Maha Sarakham, Kalasin, and Nakhon Phanom Provinces exhibit the highest prevalence (Fig. 3C). Furthermore, the comorbidity OVCCA is prominently present in Nong Bua Lamphu, Maha Sarakham, Surin, Srisaket, and Nakhon Phanom Provinces (Fig. 3D). Additionally, DMCCA is notably prevalent in Nong Bua Lamphu, Maha Sarakham, Kalasin, Surin, and Nakhon Phanom Provinces (Fig. 3E). Lastly, OVDMCCA is most prevalent in Nong Bua Lamphu, Maha Sarakham, Kalasin, and Chaiyaphum Provinces (Fig. 3F).

### Spatial poisson regression analysis

**Figure 4:**
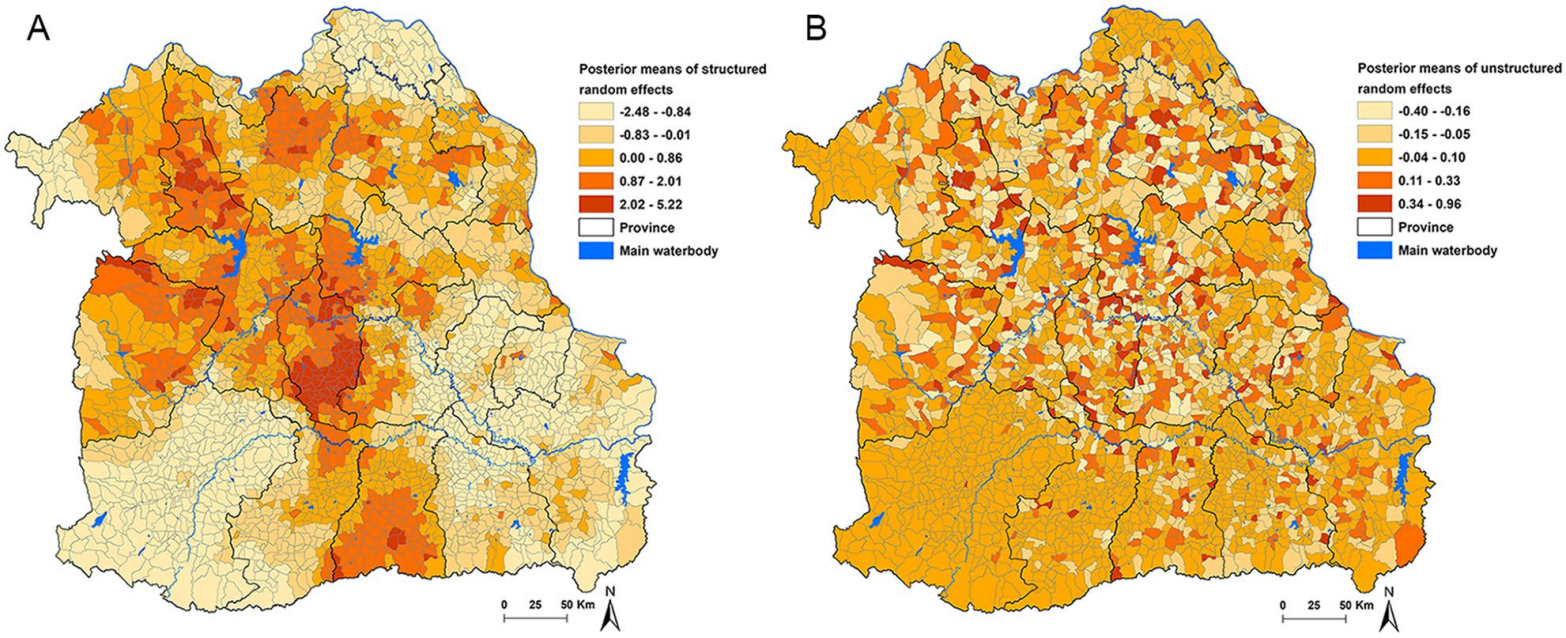
Spatial distributions of the posterior means of cholangiocarcinoma in Northeast Thailand in Model III: (**A**) Spatially structured random effects and (**B**) Spatially unstructured random effects. Maps were created using ArcGIS Pro software version 3.2 (ESRI: https://www.esri.com/en-us/home).

Table 3 presents the Bayesian spatial and non-spatial models applied to CCA. Based on the DIC statistics, the most fitting model was identified as Model III, incorporating both unstructured and structured random effects. Within Model III, gender, age, *O. viverrini* infection history, and DM exhibited significant associations with CCA. Males were found to be 2.52 times more likely to have CCA compared to females (95% CrI: 2.20–2.90). Individuals aged over 60 years had a 2.61 times higher likelihood of having CCA than those aged 60 years or younger (95% CrI: 2.28–2.98). The presence of *O. viverrini* infection history was associated with an increased risk of CCA (mean posterior relative risks (RRs) = 1.01; 95% CrI: 1.00–1.02), as was the case for DM patients (RRs = 1.02; 95% CrI: 1.01–1.03). Figure 4A showed spatial clustering was evident when accounting for the model covariates, as illustrated in the map depicting the posterior means of the spatially structured random effects. Specifically, Maha Sarakham Province, situated between the Chi and Mun rivers, exhibited a significant cluster of CCA. Other provinces, including Nong Bua Lamphu, Khon Kaen, Kalasin, Udon Thani, Surin, Nakhon Phanom, Sakon Nakhon, and Chiyaphum, also showed areas of elevated CCA risk, although these clusters were more localized and included some high-risk sub-districts. The unstructured random effects displayed the expected random spatial pattern. As expected, the map displaying the posterior means of unstructured random effects, which characterized the spatially random effects of unmeasured risk factors, showed no discernible geographic pattern (Fig. 4B).

**Table 3:** Regression coefficients, relative risks, and 95% credible interval from Bayesian models for cholangiocarcinoma in Northeast Thailand.

Model/variables	Coefficient, posterior mean (95% CrI)	RRs, posterior mean (95% CrI)
Model I (Unstructured)		
α (Intercept)	− 7.03 (− 7.22, − 6.84)	
Gender^1^	0.94 (0.80, 1.08)	2.56 (2.23, 2.94)
Age^2^	0.99 (0.85, 1.12)	2.68 (2.35, 3.06)
*O. viverrini* infection history	0.01 (0.00, 0.02)	1.01 (1.00, 1.02)
DM	0.03 (0.02, 0.03)	1.03 (1.02, 1.03)
Heterogeneity		
Structured (variance)		
Unstructured (variance)	0.44 (0.36, 0.53)	
DIC	5856.58	
Model II (Structured)		
α (Intercept)	− 7.09 (− 7.30, − 6.90)	
Sex^1^	0.93 (0.79, 1.06)	2.52 (2.20, 2.90)
Age^2^	0.96 (0.83, 1.09)	2.61 (2.28, 2.98)
*O. viverrini* infection history	0.01 (0.00, 0.02)	1.01 (1.00, 1.02)
DM	0.02 (0.01, 0.03)	1.02 (1.01, 1.03)
Heterogeneity		
Structured (variance)	0.17 (0.14, 0.22)	
Unstructured (variance)		
DIC	5695.79	
Model III (Structured and unstructured)*		
α (Intercept)	− 7.13 (− 7.34, -6.92)	
Sex^1^	0.92 (0.79, 1.06)	2.52 (2.20, 2.90)
Age^2^	0.96 (0.82, 1.09)	2.61 (2.28, 2.98)
*O. viverrini* infection history	0.01 (0.00, 0.02)	1.01 (1.00, 1.02)
DM	0.02 (0.01, 0.03)	1.02 (1.01, 1.03)
Heterogeneity		
Structured (variance)	0.21 (0.15, 0.29)	
Unstructured (variance)	4.74 (1.30, 23.04)	
DIC	5673.73*	

CrI, Bayesian credible intervals; RRs, Relative risks; DIC, Deviance information criterion.

^1^Female was reference; ^2^Age ≤ 60 years was reference.

*Best fit model.

## Discussion

This study stands as a pioneering endeavor, being the first to undertake a comprehensive spatial analysis of CCA concerning its correlation with DM and *O. viverrini* infection. Notably, these health factors are recognized as significant and prevalent issues in the Northeast Thailand. By exploring the spatial dynamics of CCA in conjunction with DM and *O. viverrini*, this research contributes to a deeper understanding of the interplay between these health concerns, shedding light on potential geographic patterns and correlations that can inform targeted interventions and public health strategies in Northeast Thailand. Our study revealed an association between *O. viverrini* infection and individuals diagnosed with DM and CCA. These observed effects remained significant even after adjusting for the influence of gender and age, both identified as significant covariates in this investigation. This discovery aligns with prior research indicating that gender and age factors are linked to CCA, particularly among males and the elderly^18,26,30,31^.

The outcomes of our investigation indicate a widespread prevalence of CCA, *O. viverrini* infection, and DM across nearly all regions in northeastern Thailand. This aligns with prior research conducted in 2011, which identified a strong association between *O. viverrini* infection and DM with CCA^18^. Furthermore, a prospective follow-up study in 2020, involving *O. viverrini*-positive subjects in Thailand, revealed a significant increase in serum levels of HbA1c and HDL during the follow-up period^32^. The spatial distribution of CCA is conspicuously apparent in the upper, middle, and specific lower regions of the studied area. This distribution aligns with a previous spatial analysis of CCA in northeast Thailand conducted in 2019, which identified significant clusters of CCA in the upper part (Nong Bua Lamphu, Udon Thani, Sakon Nakhon, and Nakhon Phanom Provinces), middle part (Maha Sarakham and Khon Kaen Provinces), and lower part (Chiyaphum Province)^20^.

On the contrary, *O. viverrini* infection and DM exhibit a widespread distribution throughout the region, consistent with a study conducted in 2021 that demonstrated a high proportion of *O. viverrini* infection and DM in a high-risk group of CCA in northeastern Thailand^18^. Moreover, in our study, the spatial distribution of CCA, *O. viverrini* infection, and DM was notably elevated, particularly in proximity to main water sources such as the Mekong, Songkhram, Chi, and Mun rivers. This finding aligns with previous research associating the prevalence of *O. viverrini* with populations residing near water sources. This observed pattern corresponds with earlier investigations, such as a 2019 study that identified a large high-risk area cluster of CCA in provinces located in the Chi and Mun River Basin, with more isolated high-risk sub-districts found in provinces situated in the Mekong River Basin^20^. Additionally, a study in 2020 revealed a significant association between geographic factors and *O. viverrini* infection^33^. Furthermore, a comprehensive review in 2018, which systematically searched five international and seven Thai research databases to identify studies relevant to risk factors for CCA in the Lower Mekong Region, recommended addressing *O. viverrini* infection as a key strategy to mitigate CCA incidence in affected regions^34^.

Our findings on Bayesian spatial analysis revealed a pronounced cluster of CCA in Maha Sarakham Province, which is situated between the Chi and Mun rivers in the middle part of the region. Subsequent notable clusters were identified in Khon Kaen and Kalasin, which are also located in the middle part, as well as provinces in the upper part (Nong Bua Lamphu, Udon Thani, Nakhon Phanom, and Sakon Nakhon), and provinces in the lower part (Surin and Chiyaphum). These findings align with prior research that has similarly identified the prevalence and spatial distribution of CCA in the northeastern region of Thailand^19,20^.

One limitation of our study pertains to the data collection method for factors related to *O. viverrini* infection and DM, which relied on participant interviews without the physical examinations or diagnostic procedures. This approach introduces the potential for data reliability issues. Furthermore, this information bias could result in underestimation or overestimation of study results, potentially impacting the overall reliability of the study's conclusions. However, the substantial size of our study's sample mitigates this concern, as the large sample size contributes to reduced variance in the data. Consequently, this diminishes the likelihood of estimation errors and strengthens the reliability of our findings, promoting more conclusive and robust outcomes.

## Conclusions

Our findings elucidate a pervasive distribution of CCA, *O. viverrini* infection, and DM across the entirety of the northeastern region of Thailand, exhibiting a congruent spatial pattern. Specifically, regions characterized by a high distribution of CCA often coincide with elevated occurrences of both *O. viverrini* infection and DM. Consequently, prioritizing CCA screening initiatives in areas exhibiting high rates of *O. viverrini* infection and prevalent DM should be accorded top priority. This targeted approach aligns with the observed spatial relationships, optimizing resource allocation for effective and region-specific health interventions.

### Supplementary Information

Supplementary Information 1.

Supplementary Information 2.

## Data Availability

The datasets generated during and/or analyzed during the current study are available from the corresponding author on reasonable request.
