# Supplementary figures and images for: Spatial analysis of cholangiocarcinoma in relation to diabetes mellitus and Opisthorchis viverrini infection in Northeast Thailand
Source: Sci Rep. 2024 May 7;14:10510. doi: 10.1038/s41598-024-61282-1 (PMC11076619; doi:10.1038/s41598-024-61282-1)

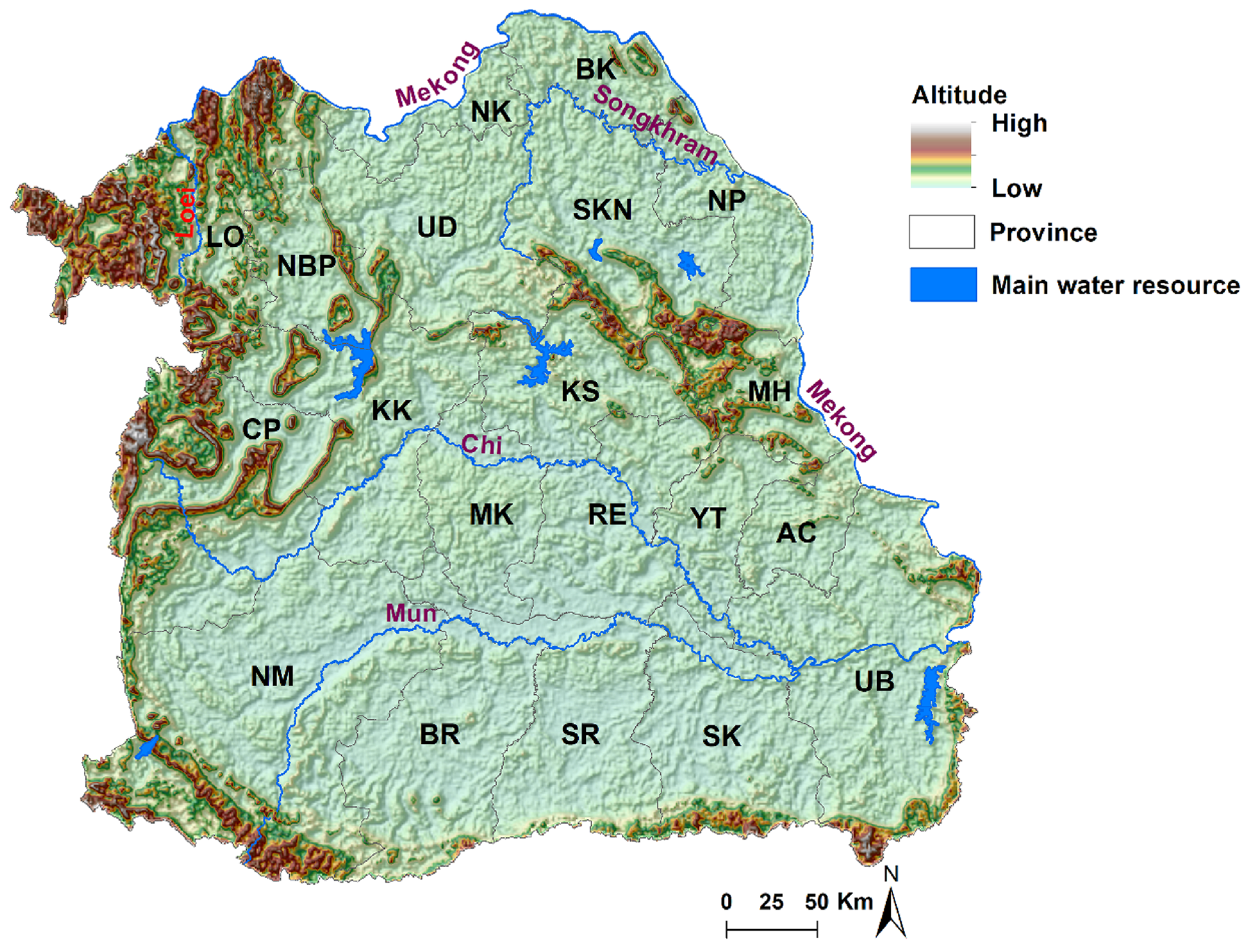

Supplement: Supplementary file 1 — Supplementary Information 1. [file 41598_2024_61282_MOESM1_ESM.tif]
