# Supplementary material for: Spatial analysis of cholangiocarcinoma in relation to diabetes mellitus and Opisthorchis viverrini infection in Northeast Thailand
Source: Sci Rep. 2024 May 7;14:10510. doi: 10.1038/s41598-024-61282-1 (PMC11076619; doi:10.1038/s41598-024-61282-1)
Supplement: Supplementary file 2 — Supplementary Information 2. [file 41598_2024_61282_MOESM2_ESM.docx]

**Supplementary figure**

**Supplementary Figure 1.** Provinces: AC - Amnat Charoen; BK - Bueng Kan; BR – Buriram; CP – Chaiyaphum; KS – Kalasin; KK - Khon Kaen; LO – Loei; MH – Mukdahan; MK - Maha Sarakham; NP - Nakhon Phanom; NR - Nakhon Ratchasima; NBP - Nong Bua Lamphu; NK - Nong Khai; RE - Roi Et; SKN - Sakon Nakhon; SK – Sisaket; SR – Surin; UB - Ubon Ratchathani; UD - Udon Thani; YT - Yasothon. Map was created using ArcGIS Pro software version 3.2 (ESRI: https://www.esri.com/en-us/home).
